# Associations between Traffic Noise, Particulate Air Pollution, Hypertension, and Isolated Systolic Hypertension in Adults: The KORA Study

**DOI:** 10.1289/ehp.1306981

**Published:** 2014-03-06

**Authors:** Wolfgang Babisch, Kathrin Wolf, Markus Petz, Joachim Heinrich, Josef Cyrys, Annette Peters

**Affiliations:** 1 Department of Environmental Hygiene, Federal Environment Agency, Berlin, Germany; 2 Institute of Epidemiology II, Helmholtz Zentrum München, Neuherberg, Germany; 3 ACCON GmbH, Greifenberg, Germany; 4 Institute of Epidemiology I, Helmholtz Zentrum München, Neuherberg, Germany; 5 Environment Science Center, University of Augsburg, Augsburg, Germany

## Abstract

Background: Studies on the association between traffic noise and cardiovascular diseases have rarely considered air pollution as a covariate in the analyses. Isolated systolic hypertension has not yet been in the focus of epidemiological noise research.

Methods: The association between traffic noise (road and rail) and the prevalence of hypertension was assessed in two study populations with a total of 4,166 participants 25–74 years of age. Traffic noise (weighted day–night average noise level; *L*_DN_) at the facade of the dwellings was derived from noise maps. Annual average PM_2.5_ mass concentrations at residential addresses were estimated by land-use regression. Hypertension was assessed by blood pressure readings, self-reported doctor-diagnosed hypertension, and antihypertensive drug intake.

Results: In the Greater Augsburg, Germany, study population, traffic noise and air pollution were not associated with hypertension. In the City of Augsburg population (*n* = 1,893), where the exposure assessment was more detailed, the adjusted odds ratio (OR) for a 10-dB(A) increase in noise was 1.16 (95% CI: 1.00, 1.35), and 1.11 (95% CI: 0.94, 1.30) after additional adjustment for PM_2.5_. The adjusted OR for a 1-μg/m^3^ increase in PM_2.5_ was 1.15 (95% CI: 1.02, 1.30), and 1.11 (95% CI: 0.98, 1.27) after additional adjustment for noise. For isolated systolic hypertension, the fully adjusted OR for noise was 1.43 (95% CI: 1.10, 1.86) and for PM_2.5_ was 1.08 (95% CI: 0.87, 1.34).

Conclusions: Traffic noise and PM_2.5_ were both associated with a higher prevalence of hypertension. Mutually adjusted associations with hypertension were positive but no longer statistically significant.

Citation: Babisch W, Wolf K, Petz M, Heinrich J, Cyrys J, Peters A. 2014. Associations between traffic noise, particulate air pollution, hypertension, and isolated systolic hypertension in adults: the KORA Study. Environ Health Perspect 122:492–498; http://dx.doi.org/10.1289/ehp.1306981

## Introduction

Environmental noise is a psychological and physiological stressor that can be an annoyance and can affect subjective well-being and physical health (van Kamp et al. 2012). Short-term exposure to continuous noise (e.g., road traffic noise) or single noise events (e.g., aircraft noise) has been shown to affect the endocrine and autonomous nervous systems in awake and in sleeping subjects (Basner et al. 2013; Ising and Kruppa 2004). Increases of blood pressure in acute noise exposure conditions have long been shown in acute noise experiments (Lehmann and Tamm 1956; Lusk et al. 2004). A meta-analysis of 24 cross-sectional studies on the association between road traffic noise and the prevalence of hypertension generated a pooled estimate of the adjusted odds ratio (OR) of 1.07 (95% CI: 1.02, 1.12) for a 10-dB(A) increase in the average noise level during the day (*L*_Aeq16hr_, at the most exposed facade) within the range of 45–75 dB(A) (van Kempen and Babisch 2012). Another meta-analysis of aircraft noise and hypertension also reported a positive association [OR = 1.13; 95% CI: 1.00, 1.28 for a 10-dB(A) increase in average day–night noise level (*L*_DN_) range, 48–68 dB(A)] (Babisch and van Kamp 2009).

Laboratory and field studies on short-term changes of blood pressure readings (day-to-day variations) carried out in recent years have been inconsistent (Brook and Rajagopalan 2009), with some reporting positive associations between particulate air pollutants and blood pressure (Brook et al. 2002, 2011) and others reporting inverse associations (Ibald-Mulli et al. 2004). Epidemiological studies of associations between long-term air pollutant exposures and blood pressure have reported positive associations (Auchincloss et al. 2008; Fuks et al. 2011) and also null or inverse associations (O’Neill et al. 2011; Sørensen et al. 2012). Newer noise studies that have accounted for nitrous gases or particulate matter as potential confounders suggest that associations of blood pressure with noise and air pollution may largely be independent of one another (de Kluizenaar et al. 2007; Dratva et al. 2012; Fuks et al. 2011; Sørensen et al. 2011b).

Isolated systolic hypertension has not yet been in the focus of epidemiological noise research. It is regarded as a risk factor for cardiovascular events on its own, particularly in the elderly (Staessen et al. 1997). It has been associated with an increased risk for heart attack and stroke (Chobanian 2007; Perry et al. 2000). In a large cohort study, the incidence of stroke was found to be associated with road traffic noise (Sørensen et al. 2011a). Anxiety, stress, and other mental strain can affect systolic blood pressure and long-term resting blood pressure (Carroll et al. 2011). Isolated systolic hypertension could reflect the effect of increased peripheral resistance (Neus et al. 1980; Sawada 1993) and arterial stiffness (Chobanian 2007; Smulyan and Safar 2000) due to stress before arteriosclerosis becomes manifest.

Within the framework of the collaborative KORA (Cooperative health research in the Region of Augsburg) health surveys, we investigated the association between exposure to road traffic noise and hypertension, taking into consideration the residential exposure of the study participants to fine particles. The KORA studies were approved by the ethics committee of the Bavarian Chamber of Physicians (Munich, Germany), and written informed consent was provided by all study participants.

## Methods

*Sample*. Since 1984, the Helmholtz Zentrum München (formerly GSF Research Center for Environment and Health) has been carrying out population studies in the region of the German city of Augsburg (inhabitants *n* = 268,896 in 2000) and the adjacent districts of Augsburg and Aichach-Friedberg (here, “Greater Augsburg”) to monitor trends and determinants in cardiovascular disease. The cross-sectional analyses reported in this article refer to the KORA-Survey 2000 S4, which was carried out from October 1999 through April 2001 by the Helmholtz Zentrum München (Holle et al. 2005). The source population comprised all German citizens 25–74 years of age whose main residence was in the City of Augsburg or in Greater Augsburg. The study population (*n* = 4,261) was a stratified random sample based on age (10-year blocks), sex, and region, including 2,090 men and 2,171 women: 1,933 from the City of Augsburg and 2,328 from Greater Augsburg. The response rate (number of participants/number eligible) was 67% (see Supplemental Material, Table S1, for additional information). The study participants were invited to temporary clinical centers for the collection of medical and questionnaire data.

*Hypertension*. Blood pressure (BP) measurements were carried out using an automatic oscillometric device (Omron Type HEM-705CP). Three blood pressure measurements were taken during the clinical interview after approximately half an hour at a 3-min interval. The average readings of the second and third measurement were considered for the analyses. Systolic/diastolic blood pressure readings ≥ 140/90 mmHg were classified as hypertensive according to guidelines [WHO (World Health Organization) and ISH (International Society of Hypertension) Writing Group 2003]. During the interview, the participants were asked whether a doctor had ever diagnosed high blood pressure and whether they take antihypertension medication. The subjects had to bring all the medication that they regularly took. Based on the medications and ATC coding (anatomical therapeutic chemical classification system), antihypertension treatment was verified. If the subjects took antihypertension medication (as an unknown side effect) but had not reported being doctor diagnosed for hypertension, they were not classified as such. Participants were classified as having prevalent hypertension based on self-reported doctor-diagnosed hypertension, measured blood pressure ≥ 140/90 mmHg, or use of antihypertension medication in conjunction with self-reported doctor diagnosed hypertension, as in previous studies (Jarup et al. 2008). Isolated systolic hypertension was defined as systolic blood pressure ≥ 140 mmHg and diastolic blood pressure < 90 mmHg in participants not being treated for high blood pressure.

*Noise assessment in the City of Augsburg*. The assessment of traffic noise in the City of Augsburg is based on the official noise map made available by the city authorities and comprised the total noise level due to road and rail traffic [Noise and Air pollution Information System (Lärm- und Luftschadstoff-Informations-System der Stadt Augsburg; LLIS) for Augsburg (ACCON Environmental Consultants 2000)]. Road noise levels were calculated according to the German standard RLS 90 (RLS90 1990), railways noise levels were calculated according to the German standard Schall 03 (Schall03 1990). The traffic data used in this study refer to the year 2001, because they reflect best the historic noise exposure of the study subjects for the time when the health assessment was carried out. The noise map has been updated since then, using traffic data from 2009 (LLIS 2013). Comparison between the noise data from 2001 and 2009 were made for validation purpose. The noise prediction software CADNA/A was used for the calculations of noise levels (DataKustik GmbH, Greifenberg, Germany). The noise propagation modeling including a three-dimensional geographic information system (GIS) considering the topography of the area (shielding due to obstacles, sound reflections). All noise levels were allocated to the geocoded addresses of the study subjects and calculated with respect to the most exposed facade of the buildings. The reference height was 4 m. Annual average equivalent A-weighted sound pressure levels were calculated for daytime (0600–2200 hours)—*L*_Aeq16hr_—and nighttime (2200–0600 hours)—*L*_Aeq8hr_. For the statistical analyses, the 24-hr weighted day–night noise indicator *L*_DN_ [penalty of 10 dB(A) for the night] was calculated, which was commonly used in noise mapping (see Supplemental Material, pp. 3–4, for additional information).

*Noise assessment in Greater Augsburg*. Calculations of traffic noise levels in Greater Augsburg were carried out within the framework of our study using the same methods as for the City of Augsburg (ACCON Environmental Consultants 2002). The data, however, which also refer to the year 2001, were less accurate. Traffic counts were available only for the superior roads, not for the inferior road network. No topographical terrain information was considered, which meant that possible shielding due to the houses themselves and other buildings could not be taken into account (free sound propagation). The noise exposure of subjects who lived in side streets was globally considered as being < 50/40 dB(A) day/night. The grid-size of the road noise calculations was 10 × 10 m with a reference level of 6 m in height (see Supplemental Material, pages 3–4, for additional information). Exposure misclassification was much more likely in Greater Augsburg. For example, the exposure of dwellings in the second row of houses could have been been overestimated due to the shielding of houses in the first row. The exposure of houses further away from the major roads could have been underestimated due to local traffic or bad road surface.

*Disentangling noise sources*. The contribution of aircraft noise was classified as insignificant and was not further considered in the analyses. The 2001 noise data did not distinguish explicitly between noise from road and from railways; only total noise levels were available. We therefore developed a method to identify participants for whom railway noise was potentially the dominant noise source outside the dwellings, and applied this method to the 2001 noise data. We used the 2009 noise maps of the City of Augsburg for this purpose. Here, separate data were available for road and railway noise (see Supplemental Material, pp. 4–5, for additional information). We estimated that railway noise was the dominant noise source for 25.2% of the participants in the City of Augsburg and 16.3% of the participants in Greater Augsburg, compared with road noise. We used this information for sensitivity analyses (excluding participants potentially exposed to railway noise).

*Air pollution*. Estimates of modeled annual average mass concentration of particles < 2.5 μm in size (PM_2.5_) at residential addresses were used as a biologically relevant indicator of exposure to air pollutants because it has been shown to be associated with the investigated end point (Brook and Rajagopalan 2009; Brook et al. 2002). The data were derived from land-use regression (LUR) models developed as part of the collaborative European Study of Cohorts for Air Pollution Effects (ESCAPE) (Eeftens et al. 2012a; ESCAPE 2013). The PM measurements were taken at 20 sites in the cities of Augsburg and Munich over three 14-day periods spread over 1 year from 2008 to 2009, using Harvard impactors (Eeftens et al. 2012b). Annual averages were calculated by adjusting for temporal variations using measurements obtained from a reference site located in urban background. A LUR model was built by combining the annual averages with geographic predictors from GIS at the monitoring sites. Individual concentrations were then estimated by applying the LUR model to the residences of the participants.

*Length of residence*. Mobility was considered as a potential factor of differential exposure misclassification. We assessed length of residence by questionnaire when the subjects came to visit the clinics. We used length of residence (adjusted for age) for sensitivity analyses, restricting the analyses to subjects that had lived in their homes for > 10 or ≤ 10 years.

*Covariates*. To adjust for potential confounding we considered the following covariates *a priori* in the statistical analyses: age (continuous), sex (men, women), smoking (current, occasionally cigarettes or < 1 cigar or pipe per day, former, never), alcohol consumption (none, < 20, 20 to < 40, 40 to < 60, 60 to < 80, ≥ 80 g/day, based on reported weekly intake of alcoholic beverages), body mass index (BMI; < 18.5, 18.5 to < 25, 25 to < 30, 30 to < 35, 35 to < 40, ≥ 40 kg/m^2^), physical activity (regularly > 2 hr/week, regularly ~ 1 hr/week, irregular ~ 1 hr/week, nearly no sport activities, based on a combination of sport activities during summer and winter), and socioeconomic status [SES; quintiles of the Helmert Index (Helmert and Shea 1994), based on school education, professional status, family income]. We also considered the percentage of households with < 1.250¤ income/month within 5 × 5 km grids as an additional index of socioeconomic deprivation in the analyses. Furthermore, based on the clinical interview, participants with a positive history of angina pectoris [Rose questionnaire (Rose 1962)] and myocardial infarction (hospital admitted) were identified.

*Statistical analyses*. Because the two samples differed considerably in the quality of noise assessment, we had decided *a priori* to carry out separate analyses within the City of Augsburg and Greater Augsburg. Because of the larger degree of exposure misclassification in Greater Augsburg, heterogeneity in the results was to be expected. However, we also carried out pooled analyses. To assess interaction, we calculated a full model including the two main factors (noise, PM_2.5_), region, and the two interaction terms of noise and PM_2.5_ with region. To assess differences between the two study samples we applied chi-square test and Mann–Whitney *U*-test statistics. Nonparametric correlation coefficients (*r*_s_) were calculated to assess associations between exposure variables. For better comparison of the results of different models, we restricted all analyses to 97.8% of the subjects with complete data for covariates, exposure, and outcome variables (City of Augsburg: *n* = 1,893; Greater Augsburg: *n* = 2,273). We adjusted for the set of basic potentially confounding covariates (age, sex, smoking, alcohol intake, BMI, SES) and additionally for PM_2.5_ according to the study hypothesis and the biological rationale. We carried out unconditional multiple logistic regression analyses using the statistical software package SPSS (version 19.0). ORs and 95% CIs were calculated. The unit scale here was 10 dB(A) for noise and 1 μg/m^3^ air pollutants, given the range of the data. For the graphical presentation of the results the noise level was categorized in 5-dB(A) categories using ≤ 45 dB(A) as a reference category [noise level categories: ≤ 45, 46–50, 51–55, 56–60, 61–65, ≥ 66 dB(A)]. Such 5-dB(A) categories with their bounds are commonly used in noise effects research (de Kluizenaar et al. 2007; Fuks et al. 2011; Jarup et al. 2008; Sørensen et al. 2011a, 2011b) and have been considered as cut points for guideline values (WHO 1999). Furthermore subgroups potentially not exposed to railway noise and with longer (> 10 years) or shorter (≤ 10 years) residence times were considered for sensitivity analyses. Statistical significance was based on an alpha level of 0.05 (*p* < 0.05, lower confidence bound > 1).

## Results

*Study characteristics*. The two samples did not statistically differ with respect to age, sex, alcohol consumption, physical activity, or the prevalence of angina pectoris or myocardial infarction (Tables 1 and 2). However, the samples differed significantly with respect to smoking habits, BMI, length of residence, and social indices. In the City of Augsburg the percentage of smokers was larger. In Greater Augsburg the BMI was slightly higher and the subjects had lived longer in their dwellings, on average. Although the area indicator of socioeconomic deprivation revealed a lower social gradient in the City of Augsburg, the individual social class indicator pointed into the opposite direction of a higher socioeconomic status in the City of Augsburg. Hypertension and isolated systolic hypertension were more prevalent in Greater Augsburg. The average noise level *L*_DN_ and the average mass concentration of PM_2.5_ were significantly higher in the City of Augsburg. The noise level *L*_DN_ and the mass concentration of PM_2.5_ were little correlated (*r*_s_ = 0.28 in both subsamples). In the City of Augsburg, the correlation was higher in the subsample potentially not exposed to railway noise (City of Augsburg *r*_s_ = 0.41, Greater Augsburg *r*_s_ = 0.29). The correlation between the weighted day–night noise level *L*_DN_ and the day noise level *L*_Aeq16hr_ was high in both samples (*r*_s_ = 0.97 and 0.98, respectively). In 2009 the calculated noise levels *L*_DN_ at the participant’s addresses of the city of Augsburg were 1.1 ± 4.3 dB(A) higher than in 2001, on average, indicating only a minor change over the years. The correlation was *r*_s_ = 0.82.

**Table 1:** Characteristics of the City of Augsburg (*n* = 1,893) and Greater Augsburg (*n* = 2,273): categorical variables.

Variable	City of Augsburg [*n* (%)]	Greater Augsburg [*n* (%)]	Chi-square test *p*-value
Sex
Women	951 (50.2)	1,169 (51.4)	0.455
Men	942 (49.8)	1,104 (48.6)
Smoking
Regular smoker	502 (26.5)	444 (19.5)	0.000
Occasional smoker	70 (3.7)	62 (2.7)
Former smoker	594 (31.4)	711 (31.3)
Never smoker	727 (38.4)	1,056 (46.5)
Alcohol consumption (g/day)
None	513 (27.1)	637 (28.0)	0.605
> 0 to ≤ 20	762 (40.3)	905 (39.8)
> 20 to ≤ 40	367 (19.4)	439 (19.3)
> 40 to ≤ 60	150 (7.9)	193 (8.5)
> 60 to ≤ 80	55 (2.9)	60 (2.6)
> 80	46 (2.4)	39 (1.7)
BMI (kg/m^2^)
< 18.5	18 (1.0)	8 (0.4)	0.004
≥ 18.5 to 25.0	666 (35.2)	714 (31.4)
≥ 25.0 to 30.0	791 (41.8)	986 (43.4)
≥ 30.0 to 35.0	315 (16.6)	419 (18.4)
≥ 35.0 to 40.0	72 (3.8)	115 (5.1)
≥ 40.0	31 (1.6)	31 (1.4)
Physical activity (hr/week)
> 2	386 (20.4)	459 (20.2)	0.324
~ 1	532 (28.1)	655 (28.8)
Occasional 1	309 (16.3)	410 (18.0)
None or very little	666 (35.2)	749 (33.0)
SES [points (quintile)]^*a*^
1–9 (low)	370 (19.5)	544 (23.9)	0.001
10–12	344 (18.2)	420 (18.5)
13–15	409 (21.6)	507 (22.3)
16–19	402 (21.2)	434 (19.1)
> 19 (high)	368 (19.4)	368 (16.2)
Length of residence (years)
≤ 10	956 (51.7)	890 (40.8)	0.000
> 10	984 (48.3)	1,289 (59.2)
Railway noise (estimated)
No	1,415 (74.7)	1,905 (83.8)	0.000
Yes	478 (25.3)	368 (16.2)
Angina pectoris
No	1,808 (95.5)	2,168 (95.4)	0.940
Yes	85 (4.5)	104 (4.6)
Myocardial infarction
No	1,856 (98.0)	2,223 (97.8)	0.664
Yes	37 (2.0)	50 (2.2)
Hypertension^*b*^
No	1,222 (64.6)	1,392 (61.2)	0.029
Yes	671 (35.4)	881 (38.8)
Isolated systolic hypertension^*c*^
No	1,469 (91.6)	1,686 (89.3)	0.021
Yes	134 (8.4)	202 (10.7)
Traffic noise [dB(A)]
≤ 45	73 (3.9)	195 (8.6)	0.000
46–50	373 (19.7)	444 (19.5)
51–55	670 (35.4)	612 (26.9)
56–60	319 (16.9)	578 (25.4)
61–65	171 (9.0)	330 (14.5)
≥ 66	287 (15.2)	114 (5.0)
^***a***^The Helmert Index is based on school education, professional status, family income. ^***b***^Prevalence of hypertension was based on self-reported doctor diagnosed hypertension or measured blood pressure ≥ 140/90 mmHg or use of antihypertension medication in conjunction with self-reported doctor-diagnosed hypertension. ^***c***^Isolated systolic hypertension was defined as systolic BP ≥ 140 mmHg and diastolic BP < 90 mmHg.			

**Table 2:** Characteristics of the City of Augsburg (*n* = 1,893) and Greater Augsburg (*n* = 2,273): continuous variables.

Variable	City of Augsburg			Greater Augsburg			*p*-Value^*a*^
	Mean ± SD	Median (IQR)	Range	Mean ± SD	Median (IQR)	Range	
Age (years)	49.0 ± 13.9	49 (24)	25–74	49.4 ± 13.8	50 (24)	25–74	0.437
BMI (kg/m^2^)	26.9 ± 4.8	26.3 (6.0)	15.8–55.1	27.5 ± 4.6	27.0 (5.8)	15.9–49.9	0.000
Length of residence (years)	14.3 ± 13.1	10 (19)	1–71	18.2 ± 15.0	15 (23)	1–74	0.000
Low-income households (%)^*b*^	42.4 ± 12.8	49.8 (8.7)	0–52.0	18.9 ± 14.6	16.1 (17.4)	0–52.0	0.000
Traffic noise level *L*_DN_ [dB(A)]	55.8 ± 7.4	54 (9.0)	36–80	54.6 ± 6.7	55 (9.0)	31–78	0.013
PM_2.5_ (μg/m^3^)	13.57 ± 0.91	13.44 (1.12)	11.77–17.68	13.71 ± 0.88	13.53 (1.10)	11.93–17.82	0.000
^***a***^Mann–Whitney *U*-test. ^***b***^Households with < 1.250€ income/month per 5 × 5 km grid.							

*Prevalence of hypertension*. The overall prevalence of hypertension was 37.3% (City of Augsburg, 35.4%; Greater Augsburg, 38.8%). Of all subjects, 6.9% were treated for hypertension and had normotensive BP readings, 9.3% were treated and had hypertensive BP readings, 9.5% were not treated (but doctor-diagnosed) and had hypertensive BP readings, and 11.5% were not aware of their high blood pressure. The overall prevalence of hypertension was higher in males (43.8%) than in females (30.9%); the proportion of subjects not aware of their high blood pressure was also higher in males (16.2%) than in females (6.9%). When 675 subjects treated for high blood pressure were excluded, the prevalence of isolated systolic hypertension in nontreated subjects was 9.6% (City of Augsburg, 8.4%; Greater Augsburg, 10.7%). Most of the covariates were significantly associated with the prevalence of hypertension (see Supplemental Material, Tables S2 and S3).

*Pooled analyses*. In the pooled sample (City of Augsburg + Greater Augsburg) the adjusted (including PM_2.5_) OR for the association between the traffic noise level and hypertension was 1.01 (95% CI: 0.90, 1.12), and for the adjusted (including noise) association between PM_2.5_ and hypertension was 1.08 (95% CI: 0.99, 1.17). The effect estimates remained nearly the same when we included the social indicator of deprivation or an indicator identifying the two samples (“region”) in the model. The interaction term for noise and region was borderline significant (*p* = 0.083), the interaction term for PM_2.5_ and region was not significant (*p* = 0.412). Similar interaction results were obtained when the social indicator of deprivation was additionally considered in the model (noise: *p* = 0.074, PM_2.5_: *p* = 0.339). Social deprivation was significantly correlated with noise (*r*_s_ = 0.23) but not with PM_2.5_ (*r*_s_ = 0.02). The results justify the *a prior*i assumption of effect modification due to differences in the quality of noise exposure assessment in the two samples (Greenland 1989; Hennekens and Buring 1987). We therefore present only stratified results in the following.

*Stratified analyses: traffic noise*. The noise level *L*_DN_ ranged between 31–80 dB(A). Table 3 shows crude and adjusted associations between the traffic noise level and the prevalence of hypertension. The crude ORs were nearly the same based on analyses of all participants and analyses limited to participants with complete data for all covariates. Noise was not significantly associated with hypertension in the Greater Augsburg population in any of the analyses. However, there was a consistent tendency of a negative association between noise and hypertension. On the other hand, we estimated significant positive associations between noise and hypertension for the City of Augsburg. Graphs of the associations in the two samples are shown in Supplemental Material, Figures S1 and S2. In the City of Augsburg higher nonsignificant ORs were estimated for all noise categories above the reference category *L*_DN_ ≤ 45 dB(A). The OR for the association between hypertension and a 10-dB(A) increase in noise in the City of Augsburg was 1.16 (95% CI: 1.00, 1.35) after adjustment for the set of covariates. After additional adjustment for PM_2.5_ the OR diminished slightly, to 1.11 (95% CI: 0.94, 1.30), and was no longer significant. Considering the prevalence of angina pectoris or myocardial infarction as additional covariates in the models did not change the noise results at all. Sensitivity analyses revealed that the adjusted OR of road traffic noise for the City of Augsburg was larger in the subgroup of 1,415 participants potentially not exposed to railway noise. In the subgroup of 894 participants with longer residence times slightly higher estimates of the relative risk were found: OR = 1.12 (95% CI: 1.12 (0.90, 1.49) after additional adjustment for PM _2.5_.

**Table 3:** Association between traffic noise (L_DN_) and the prevalence of hypertension and isolated systolic hypertension, adjusted for potentially confounding factors and air pollution (PM_2.5_).

Adjustment	City of Augsburg		Greater Augsburg	
	*n*	OR (95% CI) per 10 dB(A)	*n*	OR (95% CI) per 10 dB(A)
Hypertension
Crude	1,933	1.10 (0.98, 1.25)	2,328	1.00 (0.88, 1.13)
Crude (complete data)	1,893	1.11 (0.98, 1.26)	2,273	0.98 (0.87, 1.12)
Covariates^*a*^	1,893	1.16 (1.00, 1.35)	2,273	0.94 (0.81, 1.09)
Covariates (no railway)^*b*^	1,415	1.24 (1.04, 1.47)	1,905	0.93 (0.79, 1.09)
Covariates + PM_2.5_	1,893	1.11 (0.94, 1.30)	2,273	0.93 (0.79, 1.08)
Covariates + PM_2.5_ (no railway)^*b*^	141	1.14 (0.94, 1.39)	1,905	0.91 (0.77, 1.08)
Covariates (residence > 10 years)	894	1.19 (0.97, 1.46)	1,289	1.00 (0.84, 1.20)
Covariates (residence ≤ 10 years)	956	1.16 (1.00, 1.35)	890	0.94 (0.81, 1.09)
Covariates + PM_2.5_ (residence > 10 years)	894	1.12 (0.90, 1.40)	1,289	0.99 (0.82, 1.19)
Covariates + PM_2.5_ (residence ≤ 10 years)	956	1.11 (0.87, 1.42)	890	0.86 (0.63, 1.16)
Isolated systolic hypertension
Crude	1,601	1.38 (1.10, 1.73)	1,887	0.91 (0.73, 1.14)
Covariates^*a*^	1,601	1.48 (1.16, 1.89)	1,887	0.88 (0.69, 1.12)
Covariates + PM_2.5_	1,601	1.43 (1.10, 1.86)	1,887	0.90 (0.69, 1.15)
Covariates + PM_2.5_ (no railway)^*b*^	1,193	1.46 (1.05, 2.02)	1,590	0.89 (0.68, 1.18)
Covariates + PM_2.5_ (residence > 10 years)	682	1.18 (0.83, 1.68)	984	1.02 (0.75, 1.38)
Covariates + PM_2.5_ (residence ≤ 10 years)	878	1.68 (1.08, 2.61)	822	0.72 (0.41, 1.25)
^***a***^Adjusted for age, sex, smoking, alcohol intake, BMI, physical activity, SES. ­^***b***^Subgroup with no railway noise (­estimated).				

As for hypertension, traffic noise was not significantly associated with isolated systolic hypertension in the Greater Augsburg population, and most ORs were < 1 (Table 3). Graphs of the associations in the two samples are shown in Supplemental Material, Figures S3 and S4. In the City of Augsburg, higher nonsignificant ORs were estimated for all noise categories above *L*_DN_ = 46–50 dB(A). In the City of Augsburg (1,601 subjects) the ORs for the association between isolated hypertension and a 10-dB(A) increase in noise were considerably larger and significant (OR = 1.48; 95% CI: 1.16, 1.89, and OR = 1.43; 95% CI: 1.10, 1.86 after additional adjustment for PM_2.5_) than for hypertension. For example, the OR per interquartile range (IQR) for isolated hypertension was 1.38 (95% CI: 1.09, 1.75) after adjustment for PM_2.5_, compared with 1.10 (95% CI: 0.95, 1.27) for hypertension. The restriction to 682 participants with longer residence times diminished the OR (OR = 1.18; 95% CI: 0.83, 1.68, after adjustment for PM_2.5_), which was contradictory to the finding regarding hypertension where slightly higher ORs were found in the respective subgroup (hypertension: OR = 1.11 vs. 1.12, isolated systolic hypertension: OR = 1.43 vs. 1.18). On the other hand, the OR was much larger in the subgroup of 878 participants who had lived for shorter periods (≤ 10 years) in their homes (OR = 1.68; 95% CI: 1.08, 2.61, after adjustment for PM_2.5_).

*Stratified analyses: PM_2.5_*_._ The mass concentration of PM_2.5_ ranged between 11 and 18 μg/m^3^. As for noise, there were no significant associations between hypertension and PM_2.5_ for Greater Augsburg, and ORs were close to the null (Table 4). However, for the City of Augsburg, a 1-μg/m^3^ increase in PM_2.5_ was significantly associated with hypertension (adjusted OR = 1.15; 95% CI: 1.02, 1.30). After additional adjustment for noise, the association decreased slightly (OR = 1.11; 95% CI: 0.98, 1.27) and was no longer significant.

**Table 4:** Association between air pollution (PM_2.5_) and the prevalence of hypertension and isolated systolic hypertension, adjusted for potentially confounding factors and traffic noise (L_DN_).

Adjustment	City of Augsburg		Greater Augsburg	
	*n*	OR (95% CI) per 1 μg/m^3^	*n*	OR (95% CI) per 1 μg/m^3^
Hypertension
Crude	1,933	1.09 (0.98, 1.20)	2,328	1.06 (0.96, 1.16)
Crude (complete data)	1,893	1.08 (0.98, 1.20)	2,273	1.05 (0.96, 1.16)
Covariates^*a*^	1,893	1.15 (1.02, 1.30)	2,273	1.01 (0.91, 1.13)
Covariates + noise	1,893	1.11 (0.98, 1.27)	2,273	1.03 (0.92, 1.15)
Covariates + noise (residence > 10 years)	894	1.15 (0.97, 1.37)	1,289	1.05 (0.91, 1.21)
Covariates + noise (residence ≤ 10 years)	878	1.10 (0.90, 1.36)	822	1.04 (0.83, 1.30)
Isolated systolic hypertension
Crude	1,601	1.15 (0.96, 1.39)	1,887	0.94 (0.80, 1.12)
Covariates^a^	1,601	1.20 (0.98, 1.47)	1,887	0.94 (0.78, 1.13)
Covariates + noise	1,601	1.08 (0.87, 1.34)	1,887	0.97 (0.80, 1.17)
Covariates + noise (residence > 10 yrs)	682	1.17 (0.89, 1.56)	984	0.94 (0.75, 1.18)
Covariates + noise (residence ≤ 10 yrs)	878	1.00 (0.69, 1.45)	822	1.15 (0.77, 1.73)
^***a***^Adjusted for age, sex, smoking, alcohol intake, BMI, physical activity, SES.				

PM_2.5_ was not significantly associated with isolated systolic hypertension in Greater Augsburg (Table 4). For the City of Augsburg, the ORs were similar to those for hypertension (adjusted OR = 1.20; 95% CI: 0.98, 1.47 and adjusted OR = 1.08; 95% CI: 0.87, 1.34, after additional adjustment for noise). In contrast with the association between noise and isolated systolic hypertension, which was stronger among those with ≤ 10 years of residence than those with > 10 years of residence at the same location, the association between PM_2.5_ and isolated systolic hypertension was stronger among those with > 10 years of residence, although CIs overlapped substantially with estimates for the group with ≤ 10 years residence.

## Discussion

The hypothesis that chronic noise exposure increases the risk for cardiovascular diseases is well established. However, only a few studies have considered noise and air pollution simultaneously as potential risk factors for hypertension. We investigated the association between traffic noise and hypertension in two samples of the KORA survey, while also accounting for air pollution. We used the mass concentration of PM_2.5_ as the main representative of air pollution because particulate matter is one of the most discussed candidates with respect to cardiovascular diseases (Brook and Rajagopalan 2009; Linares et al. 2009). Both exposures were modeled with respect to the residential address of the subjects. The two samples (City of Augsburg, Greater Augsburg) differed significantly in a variety of individual subjects’ risk factors and mean exposures, and with respect to methodological aspects (noise assessment).

No significant associations with noise or air pollution were estimated for the Greater Augsburg population. In the City of Augsburg, adjusted ORs for prevalent hypertension in association with a 10-dB(A) increase in *L*_DN_ traffic noise were 1.16 (95% CI:1.00, 1.35) and 1.11 (95% CI: 0.94, 1.30) after additional adjustment for PM_2.5_. These cross-sectional results are consistent with a recent meta-analysis of 24 other cross-sectional studies on the relationship between road traffic noise and the prevalence of hypertension, which reported a pooled OR = 1.07 (95% CI: 1.02, 1.12) per increase of 10 dB(A) of the *L*_Aeq16hr_ (van Kempen and Babisch 2012). Most of the ORs included in the pooled analysis were not adjusted for air pollution. The correlation between *L*_DN_ and *L*_Aeq16hr_ was high in our study (*r*_s_ = 0.98). Associations between hypertension and noise diminished only slightly after inclusion of air pollutants as potential confounders in the model—and vice versa (in quantitative terms regardless of statistical significance). This is in line with the results of a few newer noise studies where associations with noise were found to be largely independent of the inclusion of PM_2.5_, PM_10_ (≤ 10 μm), or nitrogen dioxide (Dratva et al. 2012; de Kluizenaar et al. 2007; Sørensen et al. 2011b). Conversely, associations of PM_10_ and PM_2.5_ on blood pressure readings were found to be independent of the adjustment for traffic noise (Fuks et al. 2011).

In the City of Augsburg population—but not the Greater Augsburg population—associations with noise were much stronger for isolated systolic hypertension (systolic and diastolic blood pressure ≥ 140 mmHg and < 90 mmHg, respectively, among participants not using antihypertensive medication) than for hypertension, which was classified based on measured blood pressure (systolic blood pressure ≥ 140 and diastolic > 90 mmHg), self-reported doctor-diagnosed hypertension, and use of antihypertension medication (e.g., PM_2.5_ adjusted OR = 1.43; 95% CI: 1.10, 1.86 vs. 1.11; 95% CI: 0.94, 1.30). In contrast, associations with PM_2.5_ were similar between the two outcomes. This may have to do with different biological mechanisms of how the two agents affect the organism. According to the noise reaction model, noise stress causes vasoconstriction, which may be the predominant cause of hypertension in a shorter period of exposure. In the longer period, atherosclerosis due to metabolic changes may be more manifest. The finding that the association between noise and isolated systolic hypertension was stronger in participants that had lived for shorter periods (≤ 10 years) in their homes compared with those who had lived there for longer could reflect the short-term emotional response to the noise stress [“direct” pathway (Babisch 2002)]. Participants who had (subjectively) habituated to the noise could have developed manifest vascular changes in the longer term [“indirect” pathway (Babisch 2002)], for example, due to sleep disturbance (Basner et al. 2013). Studies carried out in schoolchildren who had not been exposed to traffic noise for long periods due to their young age also found noise effects (increases) primarily with respect to systolic blood pressure, but not for diastolic blood pressure (Paunovic et al. 2011). No such impact of length of residence on the prevalence of isolated systolic hypertension was found for air pollution.

The study has limitations. It is cross-sectional. The direction of association is not clear: Did the noise exposure precede high blood pressure or vice versa? However, it does not seem reasonable that subjects with hypertension had moved into noisy areas because of the high blood pressure. Subjects annoyed by noise (stress) may instead tend to move away from polluted areas. Our study results were inconsistent insofar as a positive association with road traffic noise was observed only in the population of the City of Augsburg, not in Greater Augsburg. However, the quality of the exposure assessment was weaker for Greater Augsburg than for the City of Augsburg. Noise levels in Greater Augsburg were only calculated for free-field noise propagation, without accounting for the shielding effect of houses or other obstacles between a major road and the subjects’ dwelling, implying a larger degree of exposure misclassification. This would be expected to bias the estimated association toward the null. Furthermore, only major roads were considered, not smaller streets near the houses that could have produced significant noise levels at the dwellings’ facades. This could explain the null findings, which were also found for air pollution in the Greater Augsburg population.

Despite the limitations, the study has several strengths. The exposure was assessed on an individual basis, and much information about potentially confounding factors was available. The correlation between noise and air pollution indicators was low, which reduced the risk of collinearity. The adjustment for air pollutants affected the effect estimates of traffic noise only slightly, and vice versa.

## Conclusions

The cross-sectional analyses of the KORA study on the association between traffic noise and hypertension revealed no significant associations with noise in the Greater Augsburg study population. However, noise was significantly associated with the prevalence of hypertension in the City of Augsburg study population. Associations with noise decreased slightly and were no longer statistically significant after adjustment for PM_2.5_. Stronger and significant associations with noise were estimated for isolated systolic hypertension compared with the composite criterion of hypertension, including blood pressure measurements, self-reported doctor-diagnosed hypertension, and use of antihypertension medication. PM_2.5_ also showed significant positive associations with the prevalence of hypertension only in the sample of the City of Augsburg; these decreased slightly and were no longer significant after adjustment for noise. Isolated systolic hypertension was not significantly associated with PM_2.5_. The null findings for the Greater Augsburg study population may be explained partly by a larger degree of exposure misclassification. The heterogeneous results between the two samples point to the need for very detailed assessments of the exposure in noise studies, because the noise level can vary considerably within short distances depending on the impact of sound attenuation due to obstacles. All in all, the results support the hypothesis that environmental noise is a risk factor for high blood pressure.

## Supplemental Material

(245 KB) PDF

## References

[r1] ACCON Environmental Consultants. (2000). Integration eines Verfahrens zur Lärmvermeidung und Luftreinhaltung in die Verwaltung und Organisation der Stadt Augsburg. Augsburg:ACCON GmbH.. http://www2.augsburg.de/fileadmin/www/dat/04um/akt_umwe/llis_endbericht.pdf.

[r2] ACCON Environmental Consultants. (2002). Kooperative Gesundheitsforschung in der Region Augsburg (KORA). Schalltechnische Untersuchung.

[r3] Auchincloss AH Diez Roux AV Dvonch JT Brown PL Barr RG Daviglus ML 2008 Associations between recent exposure to ambient fine particulate matter and blood pressure in the Multi-Ethnic Study of Atherosclerosis (MESA). Environ Health Perspect 116 486 491; 10.1289/ehp.10899 18414631 PMC2291007

[r4] Babisch W (2002). The noise/stress concept, risk assessment and research needs.. Noise Health.

[r5] Babisch W, van Kamp I (2009). Exposure-response relationship of the association between aircraft noise and the risk of hypertension.. Noise Health.

[r6] Basner M Babisch W Davis A Brink M Clark C Janssen S Stansfeld S 2013 Auditory and non-auditory effects of noise on health. Lancet; 10.1016/S0140-6736(1013)61613-X [Online 29 October 2013] PMC3988259 24183105

[r7] Brook RD, Brook JR, Urch B, Vincent R, Rajagopalan S, Silverman F (2002). Inhalation of fine particulate air pollution and ozone causes acute arterial vasoconstriction in healthy adults.. Circulation.

[r8] Brook RD, Rajagopalan S (2009). Particulate matter, air pollution, and blood pressure.. J Am Soc Hypertens.

[r9] Brook RD Shin HH Bard RL Burnett RT Vette A Croghan C 2011 Exploration of the rapid effects of personal fine particulate matter exposure on arterial hemodynamics and vascular function during the same day. Environ Health Perspect 119 688 694; 10.1289/ehp.1002107 21681997 PMC3094422

[r10] Carroll D, Phillips AC, Der G, Hunt K, Benzeval M (2011). Blood pressure reactions to acute mental stress and future blood pressure status: data from the 12-year follow-up of the West of Scotland Study.. Psychosom Med.

[r11] Chobanian AV (2007). Isolated systolic hypertension in the elderly.. N Engl J Med.

[r12] de Kluizenaar Y, Gansevoort RT, Miedema HME, de Jong PE (2007). Hypertension and road traffic noise exposure.. J Occup Environ Med.

[r13] Dratva J Phuleria HC Foraster M Gaspoz JM Keidel D Künzli N 2012 Transportation noise and blood pressure in a population-based sample of adults. Environ Health Perspect 120 50 55; 10.1289/ehp.1103448 21885382 PMC3261938

[r14] Eeftens M, Beelen R, de Hoogh K, Bellander T, Cesaroni G, Cirach M (2012a). Development of land use regression models for PM_2.5_, PM_2.5_ absorbance, PM_10_ and PM_coarse_ in 20 European study areas: results of the ESCAPE project.. Environ Sci Technol.

[r15] Eeftens M, Tsai M-Y, Ampe C, Anwander B, Beelen R, Bellander T (2012b). Spatial variation of PM_2.5_, PM_10_, PM_2.5_ absorbance and PM_coarse_ concentrations between and within 20 European study areas and the relationship with NO_2_: results of the ESCAPE project.. Atmos Environ.

[r16] ESCAPE (European Study of Cohorts for Air Pollution Effects). (2013). ESCAPE Manuals.. http://www.escapeproject.eu/manuals/index.php.

[r17] Fuks K Moebus S Hertel S Viehmann A Nonnemacher M Dragano N 2011 Long-term urban particulate air pollution, traffic noise and arterial blood pressure. Environ Health Perspect 119 1706 1711; 10.1289/ehp.1103564 21827977 PMC3261981

[r18] Greenland S (1989). Modeling and variable selection in epidemiologic analysis.. Am J Public Health.

[r19] Helmert U, Shea S (1994). Social inequalities and health status in Western Germany.. Public Health.

[r20] Hennekens CH, Buring JE. (1987). Analysis of epidemiological studies: evaluating the role of confounding: methods to control confounding in the analysis.

[r21] Holle R, Happich M, Löwel H, Wichmann HE, MONICA/KORA Study Group. (2005). KORA—a research platform for population based health research.. Gesundheitswesen.

[r22] Ibald-Mulli A Timonen KL Peters A Heinrich J Wölke G Lanki T 2004 Effects of particulate air pollution on blood pressure and heart rate in subjects with cardiovascular disease: a multicenter approach. Environ Health Perspect 112 369 377; 10.1289/ehp.6523 14998755 PMC1241869

[r23] Ising H, Kruppa B (2004). Health effects caused by noise: evidence in the literature from past 25 years.. Noise Health.

[r24] Jarup L Babisch W Houthuijs D Pershagen G Katsouyanni K Cadum E 2008 Hypertension and exposure to noise near airports: the HYENA study. Environ Health Perspect 116 329 333; 10.1289/ehp.10775 18335099 PMC2265027

[r25] Lehmann G, Tamm J. 1956 Über Veränderungen der Kreislaufdynamik des ruhenden Menschen unter Einwirkung von Geräuschen. Intern Z angew Physiol einschl Arbeitsphysiol 16:217–227. 13376178

[r26] Linares SL, Diaz J, Tobias A (2009). Are the limit values proposed by the new European Directive 2008/50 for PM_2.5_ safe for health?. Eur J Public Health.

[r27] LLIS (Lärm- und Luftschadstoff-Informations-System der Stadt Augsburg). (2013). Lärm- und Luftschadstoff Informations-System der Stadt Augsburg.. http://www.laermkarten.de/augsburg/.

[r28] Lusk SL, Gillespie B, Hagerty BM, Ziemba RA (2004). Acute effects of noise on blood pressure and heart rate.. Arch Environ Health.

[r29] Neus H, Schirmer G, Rüddel H, Schulte W (1980). On the reaction of finger pulse amplitude to noise [in German; English summary]. Int Arch Occup Environ Health.

[r30] O’Neill MS Diez-Roux AV Auchincloss AH Shen M Lima JA Polak JF 2011 Long-term exposure to airborne particles and arterial stiffness: the Multi-Ethnic Study of Atherosclerosis (MESA). Environ Health Perspect 119 844 851; 10.1289/ehp.0901524 21245016 PMC3114821

[r31] Paunovic K, Stansfeld S, Clark C, Belojevic G (2011). Epidemiological studies on noise and blood prerssure in children: observations and suggestions.. Environ Int.

[r32] Perry HM, Davis BR, Price TR, Applegate WB, Fields WS, Guralnik JM (2000). Effect of treating isolated systolic hypertension on the risk of developing various types and subtypes of stroke: the Systolic Hypertension in the Elderly Program (SHEP).. JAMA.

[r33] RLS90 (Richtlinien für Lärmschutz an Straßen-RLS 90). (1990). Richtlinien für Lärmschutz an Straßen-RLS 90. Amtsblatt des Bundesministers für Verkehr der Bundesrepublik Deutschland (VkBl.) 7 vom 14.04.1990, lfd. Nr. 70.. http://www.gesetze-im-internet.de/bimschv_16/BJNR010360990.html.

[r34] Rose GA (1962). The diagnosis of ischaemic heart pain and intermittent claudication in field surveys.. Bull World Health Organ.

[r35] Sawada Y (1993). Hemodynamic effects of short-term noise exposure.. Jpn Circ J.

[r36] Schall03. (1990). Richtlinie zur Berechnung der Schallimmissionen an Schienenwegen (Schall 03). Amtsblatt der Deutschen Bundesbahn Nr. 14 vom 4. April 1990, lfd. Nr. 133.. http://www.gesetze-im-internet.de/bimschv_16/BJNR010360990.html.

[r37] Smulyan H, Safar ME (2000). The diastolic blood pressure in systolic hypertension.. Ann Intern Med.

[r38] Sørensen M Hoffmann B Hvidberg M Ketzel M Jensen SS Andersen ZJ 2012 Long-term exposure to traffic-related air pollution associated with blood pressure and self-reported hypertension in a Danish cohort. Environ Health Perspect 120 418 424; 10.1289/ehp.1103631 22214647 PMC3295339

[r39] Sørensen M, Hvidberg M, Andersen ZJ, Norsborg RB, Lillelund KG, Jakobsen J (2011a). Road traffic noise and stroke: a prospective cohort study.. Eur Heart J.

[r40] Sørensen M Hvidberg M Hoffmann B Andersen ZJ Norsborg RB Lillelund KG 2011b Exposure to road traffic and railway noise and association with blood pressure and self-reported hypertension: a cohort study. Environ Health 10 92; 10.1186/1476-069X-10-92 22034939 PMC3213219

[r41] Staessen JA, Fagard R, Thijs L, Celis H, Arabidze GG, Birkenhäger WH (1997). Randomised double-blind comparison of placebo and active treatment for older patients with isolated systolic hypertension.. Lancet.

[r42] van Kamp I, Babisch W, Brown AL. (2012). Environmental noise and health.

[r43] van Kempen E, Babisch W (2012). The quantitative relationship between road traffic noise and hypertension: a meta-analysis.. J Hypertens.

[r44] WHO (World Health Organization). (1999). Guidelines for Community Noise.

[r45] WHO (World Health Organization) and ISH (International Society of Hypertension) Writing Group (2003). World Health Organization (WHO)/International Society of Hypertension (ISH) statement on management of hypertension.. J Hypertens.

