# Supplementary material for: Associations between Traffic Noise, Particulate Air Pollution, Hypertension, and Isolated Systolic Hypertension in Adults: The KORA Study
Source: Environ Health Perspect. 2014 Mar 6;122(5):492–8. doi: 10.1289/ehp.1306981 (PMC4014763; doi:10.1289/ehp.1306981)
Supplement: (245 KB) PDF [file ehp.1306981.s001.pdf]

Environmental Health Perspectives doi:10.1289/ehp.1306981

SUPPLEMENTAL MATERIAL

Associations between Traffic Noise, Particulate Air Pollution, Hypertension, and Isolated Systolic Hypertension in Adults: The KORA Study

Wolfgang Babisch, Kathrin Wolf, Markus Petz, Joachim Heinrich, Josef Cyrys, and Annette Peters

TABLE OF CONTENTS Page

METHODS, ADDITIONAL DESCRIPTIONS **2**

Noise assessment 2

Disentangling road and railway noise 3

TABLES 5

Table S1: Response statistic 5

Table S2: Univariate associations between covariates and the prevalence of hypertension 6

Table S3: Univariate associations between covariates and the prevalence of isolated systolic hypertension 8

FIGURES 10

Figure S1: Association between traffic noise and the prevalence of hypertension (City of Augsburg) 10

Figure S2: Association between traffic noise and the prevalence of hypertension (Greater Augsburg) 11

Figure S3: Association between traffic noise and the prevalence of isolated systolic hypertension (City of Augsburg) 12

Figure S4: Association between traffic noise and the prevalence of isolated systolic hypertension (Greater Augsburg) 13

Methods, additional descriptions

Sample selection

For the selection of study subjects a stratified random sample (quota sampling) of 6,640 subjects was drawn from the registry office (appointed date 1st June 1999). Stratification criteria were age (10 year blocks), gender and region (City of Augsburg, Greater Augsburg), resulting in 10 strata of 664 subjects. In the City of Augsburg stratified random sampling was done directly. In Greater Augsburg a two-step procedure was applied. In a first step 16 of 70 municipalities were randomly selected ("probability proportional to size"), and in a second step the random sampling of subjects was done. Table S1 shows the breakdown statistic of the response. For the age-reference the 6th of June 2000 was chosen, which was in the middle of the data collecting phase.

Noise assessment

Traffic counts including vehicle composition - as obtained from the road construction office of Augsburg, local administration offices, and the Bavarian highway department - were available for all streets in the City of Augsburg with a traffic volume of approximately 2,100 vehicles or more per 24 hours on a workday (primary road network). The inferior road network was categorized into collector roads (1000 vehicles/24 h), communicator roads between collector roads (500 vehicles/24 h), and residential streets (250 vehicles/24 h). For Greater Augsburg traffic counts were only available for the superior roads, including major trunk roads, state roads and through roads (primary road network). Based on measurements carried out in other parts of the administrative district of Schwaben, inferior roads such as district roads were globally considered with a daily traffic volume of 2,100 vehicles, including 10% heavy vehicles (59/48 dB(A) during day-time/night-time, at a 25 m distance from the road). Noise from railways was

calculated according to the German standard Schall 03. Railway traffic volume, type of train, and track speeds were obtained from the Civil Engineering Office of Augsburg and the German Railway Company ("Deutsche Bahn AG"), reference year 2001.

The geo-coded addresses of the study subjects were obtained from the cadastral land register of the City of Augsburg and the surrounding villages of Greater Augsburg. In unclear situations (e. g. missing house numbers), the addresses were visited for visual inspection. Day and night noise levels were highly correlated (City of Augsburg: $r_s = 0.93$, Greater Augsburg: $r_s = 0.95$) which is a common experience regardless of the actual 24 hour traffic volume. Consequently, the correlation between L_{DN} and L_{Aeq16h} was also high (City of Augsburg: $r_s = 0.97$, Greater Augsburg: $r_s = 0.98$). For comparison of the noise data from 2001 and 2009 the total noise level L_{DN} (road and railway traffic) was calculated from the 2009 noise maps (City of Augsburg only).

Disentangling road and railway noise

The 2001 noise data did not distinguish explicitly between the two noise sources. For sensitivity analyses (exclusion of subjects), a method was developed to identify participants where railway noise was potentially the dominant noise source at home. This was done using the City of Augsburg noise data from 2009 because separate road and railway noise levels were available from the these noise maps for day (L_{Aeq16h}) and for the night-time (L_{Aeq8h}).

Railway noise levels included the so called "railway bonus" of 5 dB(A), meaning that the railway noise levels were 5 dB(A) lower than actually measured or calculated. In a first step the 2009 railway noise levels were shifted by 5 dB(A) to eliminate the '"railway bonus"', and the differences between the road noise levels and the railway noise levels were calculated for the day and the night-time. If either the day noise level or the night noise level of the railway noise was

at least 5 dB(A) higher than the respective road noise level, the subjects were classified as predominantly railway noise exposed. This criterion was a pragmatic setting considering that a sound level difference of 3 dB(A) (sound intensity double as high) is just about audible, and that a difference of 10 dB(A) (ten-fold sound intensity) is perceived to be twice as loud. The criterion was fulfilled for 19.0% of participants of the City of Augsburg sample that had not moved on the basis of the 2009 noise data.

In the next step a method was to be found that could be applied to the 2001 noise data and that identified largely the same subjects. It is a common experience that road noise levels in urban streets (no motorways) fall by approximately 7 to 11 dB(A) during the night-time compared with the day-time. This is less the case for railway noise due to increased freight traffic during the night. This was confirmed by the 2009 noise data showing mean differences between day and night noise levels of 9 dB(A) (standard deviation SD = 1.0) dB(A) for road traffic noise, and 3 dB(A) (SD = 2.7) for railway noise, respectively. Using this information and following up the concept that the railway noise level (without railway bonus) has to be at least 5 dB(A) higher than the road noise level in order to be the dominant source, a day-night difference of 6 dB(A) or less of the 2009 total noise levels (road + rail including the railway bonus) was found to be an alternative criterion for the identification of participants that were potentially exposed to dominant railway noise. This was the case for 22.7% of participants of the City of Augsburg. The statistical sensitivity of this alternative criterion was 0.90, the specificity 0.93 (2009 data).

When this alternative criterion was applied to the 2001 noise data it was estimated that railway noise was the dominant noise source for 25.2 % and 16.3 % of participants in the City of Augsburg and Greater Augsburg, respectively.

Table S1. Response statistic.

Variable	*n*	Percent
Identified subjects	6,640	100.0%
Insufficient German language	37	0.6%
Deceased before contact	51	0.8%
Moved out of area	172	2.6%
Eligible subjects	6,380	100.0%
Not able to contact	172	2.7%
Seriously ill	199	3.1%
Subject too busy ("no time")	338	5.3%
Refused	1,410	22.1%
Participants	4,261	66.8%

Table S2. Univariate associations between covariates and the prevalence of hypertension.

Variable	City of Augsburg: OR (95% CI)	Greater Augsburg: OR (95% CI)
**Age**		
Per 1 year	1.07 (1.06, 1.08)	1.08 (1.07, 1.08)
**Gender**		
Women	1	1
Men	1.92 (1.59, 2.33)	1.62 (1.37, 1.93)
**Smoking**		
Never smoker	1	1
Former smoker	0.61 (0.47, 0.78)	0.52 (0.41, 0.66)
Occasional smoker	0.70 (0.41, 1.20)	0.54 (0.30, 0.95)
Regular smoker	1.32 (1.06, 1.65)	1.09 (0.90, 1.33)
**Alcohol**		
No alcohol consumption	1	1
>0 to ≤20 g/day	0.71 (0.56, 0.90)	0.87 (0.71, 1.07)
>20 to ≤40 g/day	0.91 (0.69, 1.20)	0.89 (0.70, 1.15)
>40 to ≤60 g/day	1.27 (0.88, 1.83)	1.36 (0.99, 1.89)
>60 to ≤80 g/day	1.99 (1.14, 3.48)	1.43 (0.84, 2.43)
>80 g/day	1.97 (1.08, 3.62)	1.45 (0.76, 2.77)
**Body mass index**		
<25 kg/m^2	1	1
≥25.0 to 30.0 kg/m^2	3.45 (2.70, 4.42)	3.26 (2.59, 4.09)
≥30.0 to 35.0 kg/m^2	6.32 (4.68, 8.53)	5.93 (4.52, 7.78)
≥35.0 to 40.0 kg/m^2	7.94 (4.75, 13.3)	9.60 (6.22, 14.8)
≥40.0 kg/m^2	7.00 (3.33, 14.7)	19.0 (7.63, 47.2)
**Physical activity**		
>2 hours/week	1	1
Ca. 1 hour/week	0.85 (0.64, 1.14)	1.07 (0.83, 1.38)
Occasional 1 hour/week	1.49 (1.09, 2.03)	1.37 (1.04, 1.81)
None or very little	1.60 (1.23, 2.08)	1.89 (1.48, 2.41)
**Socio-economic status (quintiles)**[a]		
1 to 9 points	1	1
10 to 12 points	1.09 (0.81, 1.47)	0.88 (0.68, 1.13)
13 to 15 points	0.70 (0.52, 0.94)	0.68 (0.53, 0.87)
16 to 19 points	0.72 (0.53, 0.96)	0.56 (0.43, 0.73)
>19 points	0.63 (0.46, 0.85)	0.59 (0.45, 0.77)
**Low income households**[b]		
Per % increase	1.01 (1.00, 1.02)	1.00 (1.00, 1.01)
**Residence time**		
≤10 years	1	1
>10 years	2.80 (2.30, 3.41)	2.61 (2.17, 3.14)

Variable	City of Augsburg: OR (95% CI)	Greater Augsburg: OR (95% CI)
**Residence time**		
Per 1 year	1.04 (1.04, 1.05)	1.04 (1.03, 1.05)
**Railway noise (estimated)**		
No	1	1
Yes	1.07 (0.86, 1.33)	1.00 (0.79, 1.25)
**Angina pectoris**		
No	1	1
Yes	1.36 (0.87, 2.11)	2.07 (1.39, 3.07)
**Myocardial infarction**		
No	1	1
Yes	5.87 (2.75, 12.5)	3.45 (1.89, 6.29)

[a]The "Helmert Index" is based on school education, professional status, family income.

[b]Households with less than 1.250 € income per 5 x 5 km grid.

Table S3. Univariate associations between covariates and the prevalence of isolated systolic hypertension.

Variable	City of Augsburg: OR (95% CI)	Greater Augsburg: OR (95% CI)
**Age**		
Per 1 year	1.07 (1.05, 1.07)	1.08 (1.07, 1.10)
**Gender**		
Women	1	1
Men	1.79 (1.24, 2.58)	1.94 (1.43, 2.63)
**Smoking**		
Never smoker	1	1
Former smoker	0.66 (0.40, 1.09)	0.37 (0.23, 0.60)
Occasional smoker	0.79 (0.27, 2.25)	0.65 (0.26, 1.67)
Regular smoker	1.56 (1.04, 2.33)	0.93 (0.67, 1.29)
**Alcohol**		
No alcohol consumption	1	1
>0 to ≤20 g/day	0.71 (0.45, 1.12)	0.83 (0.57, 1.20)
>20 to ≤40 g/day	0.90 (0.53, 1.52)	1.08 (0.71, 1.65)
>40 to ≤60 g/day	1.08 (0.54, 2.13)	1.11 (0.64, 1.94)
>60 to ≤80 g/day	2.82 (1.29, 6.13)	1.46 (0.65, 3.26)
>80 g/day	1.49 (0.55, 4.04)	1.70 (0.67, 4.27)
**Body mass index**		
<25 kg/m^2	1	1
≥25.0 to 30.0 kg/m^2	2.65 (1.68, 4.16)	2.22 (1.51, 3.27)
≥30.0 to 35.0 kg/m^2	3.15 (1.82, 5.44)	3.62 (2.34, 5.60)
≥35.0 to 40.0 kg/m^2	2.96 (1.08, 8.09)	2.03 (0.87, 4.75)
≥40.0 kg/m^2	4.49 (1.23, 16.4)	4.41 (1.40, 13.9)
**Physical activity**		
>2 hours/week	1	1
Ca. 1 hour/week	0.78 (0.45, 1.35)	1.02 (0.65, 1.59)
Occasional 1 hour/week	1.13 (0.63, 2.05)	1.13 (0.69, 1.85)
None or very little	1.49 (0.92, 2.42)	1.65 (1.09, 2.49)
**Socio-economic status (quintiles)**[a]		
1 to 9 points	1	1
10 to 12 points	0.85 (0.51, 1.42)	1.03 (0.68, 1.56)
13 to 15 points	0.50 (0.29, 0.86)	0.68 (0.45, 1.04)
16 to 19 points	0.60 (0.34, 0.97)	0.55 (0.35, 0.87)
>19 points	0.43 (0.24, 0.77)	0.41 (0.24, 0.70)
**Low income households**[b]		
Per % increase	1.01 (0.99, 1.02)	0.99 (0.98, 1.00)
**Residence time**		
≤10 years	1	1
>10 years	2.47 (1.67, 3.60)	2.59 (1.86, 3.62)

Variable	City of Augsburg: OR (95% CI)	Greater Augsburg: OR (95% CI)
**Residence time**		
Per 1 year	1.05 (1.04, 1.06)	1.04 (1.03, 1.05)
**Railway noise (estimated)**		
No	1	1
Yes	1.05 (0.70, 1.57)	0.93 (0.62, 1.40)
**Angina pectoris**		
No	1	1
Yes	1.61 (0.75, 3.46)	0.69 (0.27, 1.74)
**Myocardial infarction**		
No	1	1
Yes	1.71 (0.38, 7.65)	2.70 (1.07, 6.84)

[a]The "Helmert Index" is based on school education, professional status, family income. [b]Households with less than 1.250 € income per 5 x 5 km grid.

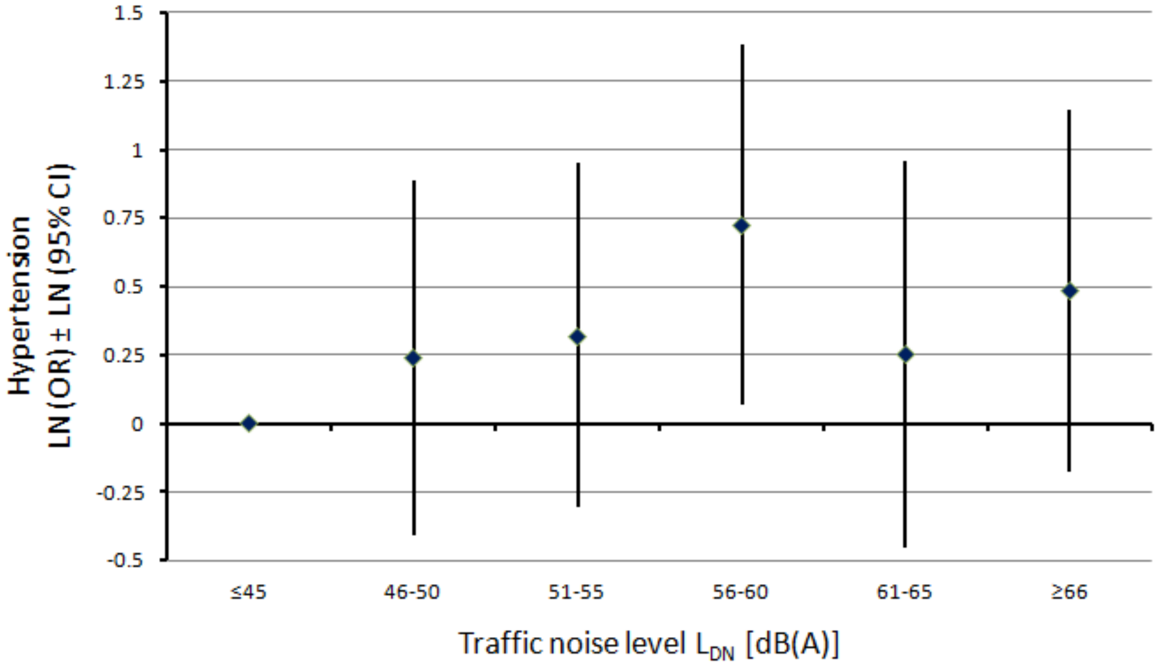

Figure S1. Association between traffic noise (noise level categories) and the prevalence of hypertension, adjusted for age, gender, smoking, alcohol intake, body mass index, physical activity, socio-economic status (City of Augsburg).

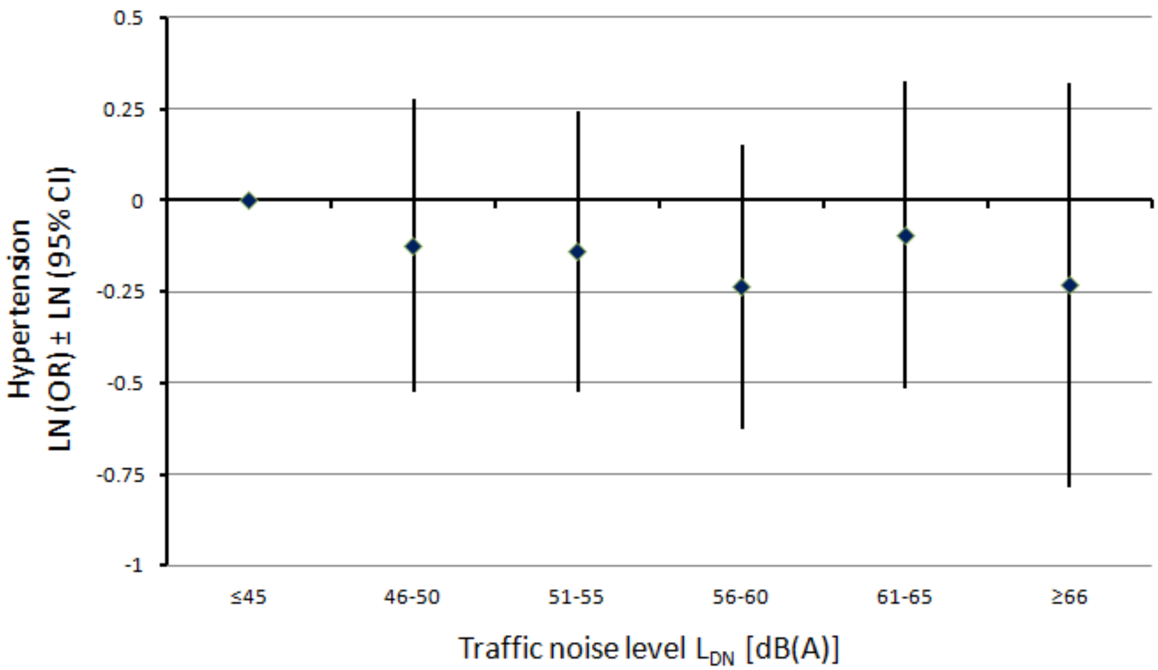

Figure S2. Association between traffic noise (noise level categories) and the prevalence of hypertension, adjusted for age, gender, smoking, alcohol intake, body mass index, physical activity, socio-economic status (Greater Augsburg).

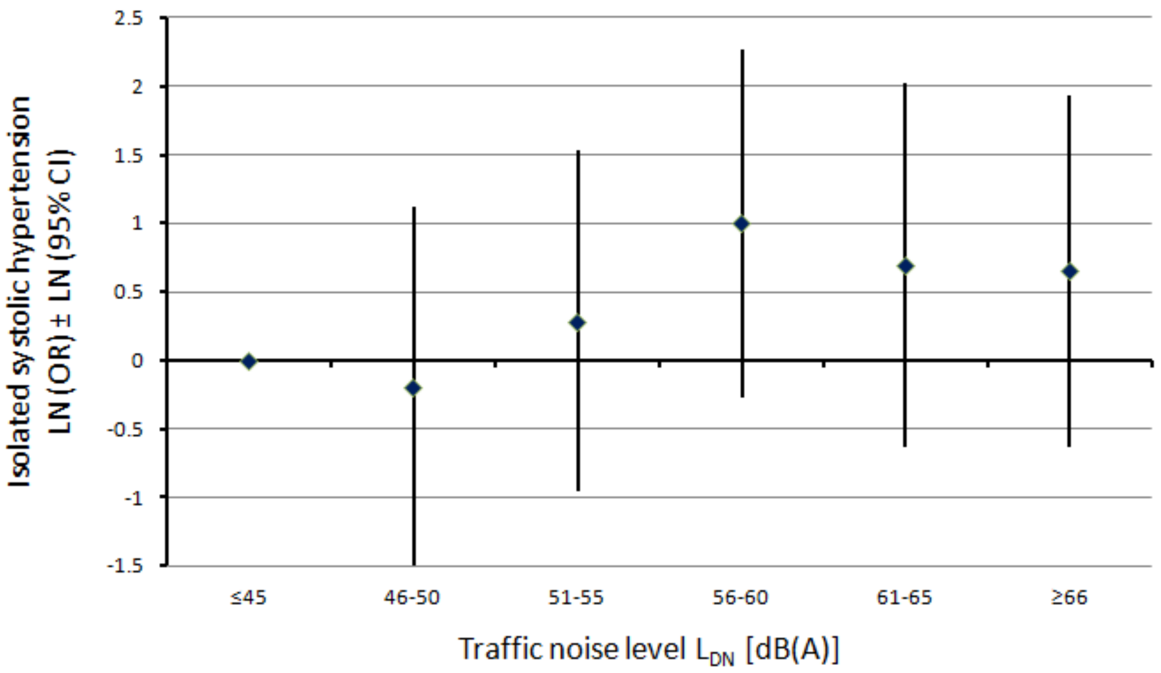

Figure S3. Association between traffic noise (noise level categories) and the prevalence of isolated systolic hypertension, adjusted for age, gender, smoking, alcohol intake, body mass index, physical activity, socio-economic status (City of Augsburg).

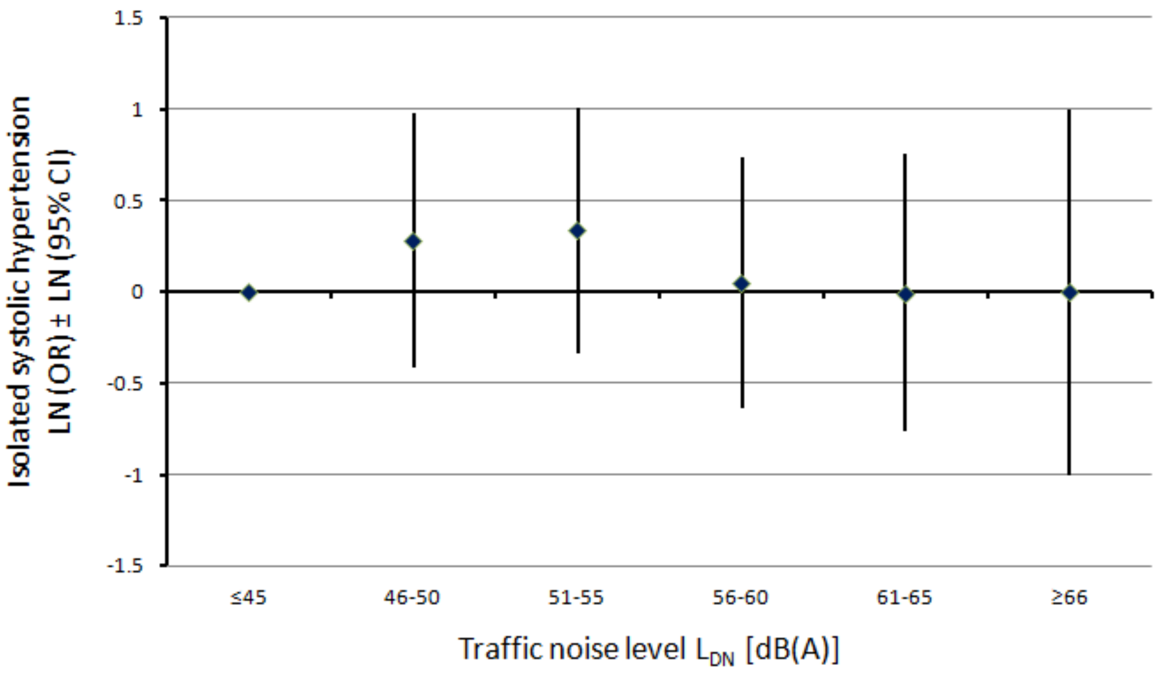

Supplemental Material, Figure S4. Association between traffic noise (noise level categories) and the prevalence of isolated systolic hypertension, adjusted for age, gender, smoking, alcohol intake, body mass index, physical activity, socio-economic status (Greater Augsburg).
